# Recognizing and responding to women experiencing homelessness with gendered and trauma-informed care

**DOI:** 10.1186/s12889-020-8353-1

**Published:** 2020-03-26

**Authors:** Katrina Milaney, Nicole Williams, Stacy Lee Lockerbie, Daniel J. Dutton, Elaine Hyshka

**Affiliations:** 1 Community Rehabilitation and Disability Studies, Cumming School of Medicine, University of Calgary, 3280 Hospital Dr NW, Calgary, AB T2N 4N1 Canada; 2 University of Calgary’s School of Public Policy, 2500 University Dr NW, Calgary, AB T2N 1N4 Canada; 3 Department of Nursing, University of Calgary, 2500 University Dr NW, Calgary, AB T2N 1N4 Canada; 4 Department of Community Health and Epidemiology at Dalhousie University, Centre for Clinical Research, 5790 University Avenue, Halifax, NS B3H 1V7 Canada; 5 School of Public Health at the University of Alberta, 3-300 Edmonton Clinic Health Academy, 11405 – 87 Ave, Edmonton, AB T6G 1C9 Canada

**Keywords:** Women, Mental health, Addiction, Trauma-informed care, Homelessness

## Abstract

**Background:**

The purpose of this study is to highlight the experiences of women who are often hidden in what we know and understand about homelessness, and to make policy and practice recommendations for women-centred services including adaptations to current housing interventions.

**Methods:**

Three hundred survey interviews were conducted with people experiencing homelessness in Calgary, Alberta, Canada. The survey instrument measured socio-demographics, adverse childhood experiences, mental and physical health, and perceived accessibility to resources. Eighty-one women participants were identified as a subsample to be examined in greater depth. Descriptive statistics and logistic regressions were calculated to provide insight into women respondents’ characteristics and experiences of homelessness and how they differed from men’s experiences.

**Results:**

Women’s experiences of homelessness are different from their male counterparts. Women have greater mental health concerns, higher rates of diagnosed mental health issues, suicidal thoughts and attempts, and adverse childhood trauma. The results should not be considered in isolation, as the literature suggests, because they are highly interconnected.

**Conclusion:**

In order to ensure that women who are less visible in their experiences of homelessness are able to access appropriate services, it is important that service provision is both gender specific and trauma-informed. Current Housing First interventions should be adapted to ensure women’s safety is protected and their unique needs are addressed.

## Background

While women made up 27% of the Canadian homeless population in 2016, 89% of families in shelters were found to be headed by women [1]. Homeless women are considered “one of the most vulnerable subpopulations among the homeless,” [2] which is attributable to many interrelated factors such as a shortage of women-centred services [3], women’s vulnerability to violence, exploitation and marginalization [4], and higher rates of poverty [5] often lacking the necessary resources when escaping violence. Evidence suggests that women are largely underrepresented in homeless counts as they are less visible in their experiences of homelessness [6, 7], .Women often seek shelter in “non-service-led situations” such as sleeping rough, couch surfing or staying in unsafe housing [8]. It has been suggested that women experience both “spatial and policy invisibility,” [9] and as a result are less likely to be effectively served by conventional health and social services. One study identified that “women as a homeless population have conventionally been left out of societal discourse, public policy, and research agendas, thereby affecting funding priorities and the development of service delivery models.” [7] Evidence also shows that without appropriate interventions, women have a high likelihood for multiple episodes of homelessness [10].

Housing First is an intervention that provides affordable housing and case management services and has been identified as a ‘best practice’ intervention that can sustainably end homelessness for people with complex physical and mental health issues. However, in a Canada wide research study to determine its effectiveness, “the ‘typical’ participant was a male in his early 40’s…” [11]. This research team concluded that further research is needed to determine how to adapt the model for particular sub-groups including women.

Much of the homelessness literature indicates that homelessness for women is unique from men’s experiences as women’s experiences are governed by oppression, gender inequality, and stigma [7, 12]. For instance, women are particularly vulnerable to victimization and are at higher risk for physical and sexual violence while living on the streets or in shelters [9, 13, 14]. Furthermore, intimate partner violence is a common path into homelessness for women [7, 12] as a lack of affordable housing in Canada and limited financial and social resources often require women to choose between staying with their abusive partner and falling into homelessness [15]. Adverse childhood experiences have been associated with concurrent mental health concerns and substance use disorders [16], frequent health care use in adulthood [17], and poor physical and mental health outcomes [18]. Physical and sexual abuse during childhood has been found to be particularly prevalent amongst women experiencing homelessness [19–21] and women disproportionately require hospital care, particularly with regards to recent violence and mental health concerns [2, 22].

Women experiencing homelessness have also been found to report high rates of gender-specific health concerns related to gynaecological health, pregnancy, feminine hygiene, and sexually transmitted infections [23, 24]. Women living on the streets are particularly vulnerable as women sleeping rough, have been found to have higher rates of poor physical and mental health issues and higher reported rates of substance use than women living in shelters [25, 26].

Women experiencing homelessness face many barriers to accessing appropriate health care services, such as long wait times, lengthy travel time, and lack of transportation [27, 28]. It has also been identified that for some women, addiction “may be overshadowing other pressing health concerns.” [29] The physical, mental, and sexual health concerns of women experiencing homelessness are complex, thus indicating that it is critical to facilitate access to appropriate and safe interventions.

In order to ensure that women can easily obtain primary, preventative, and specialized health care, access to appropriate services must be facilitated through establishing gender-sensitive and trauma-informed care [29]. However, there is little pre-existing research examining the perceived need for health care services amongst women experiencing homelessness nor is there much that has focused on adverse childhood experiences and their impact on women’s long-term health and social outcomes.

It is prudent to recognize the history of victimization and exploitation of women and how their experiences of homelessness are different than men. The needs of women must be addressed through homeless reduction strategies as gendered homelessness for women is gendered [7]. Strategies to address homelessness must adopt a gendered and trauma informed perspective.

The purpose of this study is to better understand if women are more likely to experience suicide attempts and/or psychiatric stays than men and if these experiences are influenced by mental illness and/or childhood trauma. Applying a gendered lens to our analysis meant we carefully and deliberately examined the data and its implications considering the different needs and experiences of women as compared to men. The following analysis will help fill existing gaps, providing insight and recommendations into how services and practices can be improved to respond to the gender and trauma specific needs of women. We posit suggestions for enhancements to homelessness interventions including Housing First programs that are reflective of and responsive to the unique needs and vulnerabilities of women.

## Methods

Survey interviews were conducted with 300 people experiencing homelessness in Calgary, Canada between January and March 2017. The interview guide used some of the questions from the *Perceived Need for Care Questionnaire* [30] with some additions and adaptions thought relevant by the research team. The fulsome version of the *Adverse Childhood Experiences survey* was also used [31]. Eligibility criteria required participants to have been homeless for at least 6 months or more than four times in the last 2 years. All participants provided informed consent prior to completing the survey and received a $25 (CAD) honorarium for their time. Research was conducted primarily at two emergency shelters in Calgary, and a small group (*n* = 18) of rough sleepers were also interviewed outside. Respondents were asked a series of 88 questions regarding their socio-demographics, adverse childhood experiences (the ACE survey), mental and physical health, and access to services.

The data were inputted electronically, checked for consistency and analysed using SPSS. For the purposes of our analysis, the 81 self-identified women participants comprise the subsample of respondents examined in greatest depth. When running comparative analyses, the 217 self-identified men respondents comprised the other subsample of respondents. Remaining respondents were excluded from the analysis, as the sample size was too small to examine independently. SPSS was then used to examine respondents’ demographics, adverse childhood experiences, substance use, and mental health status. Descriptive statistics and logistic regressions (adjusted and unadjusted) provide insight into women respondents’ characteristics and experiences of homelessness and how they differed from men.

### Measures

Respondents were asked to report the various places that they had slept in the past 6 months. Seven of the options provided to respondents were collapsed to create the hidden homeless score variable: sleeping in friends places, sleeping in family members’ places, couch surfing, sleeping in jail/prison, in hospitals, in vehicles, or walking all night. Respondents’ ACE scores were scores out of 10 and were based on whether or not respondents experienced the following experiences before the age of 18: verbal, physical, sexual, and emotional abuse, neglect, abandonment, witnessing a parent being abused, if a parent struggled with addiction, if a household member had a mental health issue or attempted suicide, and if a household member went to prison. The higher the number of adverse childhood experiences the participant had prior to the age of 18, the higher the ACE score.

Respondents identified the length of time and the number of times they had been homeless in their lives (once, twice, three times, four times, five times, or more than five times). These variables provide relative measures of patterns of homelessness. The same method was used to create variables for patterns of alcohol use, six or more drinks on one occasion, non-beverage alcohol consumption, drug use, polydrug use, and using both alcohol and drugs at the same time. Each of these variables were measured based on respondents’ self-report of how often each occurred: these were scored using the following scale: never, monthly or less, weekly, and daily or almost daily, which were then used to provide relative measures of substance use frequency. Respondents were asked to identify if they had any diagnosed or undiagnosed mental health issues, suicidal thoughts and attempts, and whether or not they had been in a mental health facility.

The outcome variables were selected based upon evidence that suggest these variables may impact the relationships between gender and the outcome variables of interest. For instance, an individual’s age and Indigenous identity shape their experiences and their help-seeking behaviour. The length of time experiencing homelessness may impact an individual’s likelihood to attempt suicide and/or stay in a psychiatric hospital. Further, mental illness diagnoses and higher ACE scores may impact an individual’s likelihood to require psychiatric attention or to attempt suicide.

This study received ethics approval from the University of Calgary Conjoint Health Research Ethics Board, REB17–2064.

## Results

Women were found to be significantly more likely to have stayed in a psychiatric hospital. Women were significantly more likely to have reported having suicidal thoughts in their lifetime, having attempted suicide in their lifetime, and having been diagnosed with a mental health issue. Identifying as a woman was also associated with the number of reported mental health diagnoses.

When examining associations with substance use and addiction diagnoses, women’s experiences with substance use were not significantly different than men’s; however, examining their substance use patterns provides insight into the consequences of use for women. The largest proportion of women reported using drugs other than alcohol daily or almost daily (37%), while 18.5% use drugs monthly or less and 13.5% reported using drugs weekly. Of those who use drugs, 71.6% reported never using more than one drug at the same time, 54.3% reported never using alcohol and drugs at the same time, while 22.2% reported doing so monthly or less, 13.6% did so weekly, and 9.8% did so daily or almost daily. Besides alcohol and marijuana, the most commonly reported substances used were crack (34.6% of respondents), cocaine (22.2%), and methamphetamines (21%). The consequences of using were notable as 60.5% of respondents had legal difficulties as a result of drinking or drug use and 49.4% reported that their drug use had hurt themselves or others. Additionally, 22.2% of women felt that their longing for drugs was irresistible daily or almost daily and 17.3% had guilty feelings or a bad conscience for using drugs.

Women reported disproportionately high levels of adverse childhood experiences, including notably higher rates of verbal and sexual abuse in childhood than men. These results are supplemental to the analysis using respondents’ ACE score in Table 1 that identified the statistically significant correlation between gender and the number of adverse childhood experiences.

**Table 1:** Systems Usage and Health Status

	Women (*n* = 81)					Men (*n* = 217)				
	Frequency	Mean	Median	Std. Dev.	Range	Frequency	Mean	Median	Std. Dev.	Range
Systems usage										
Stay in psychiatric hospital^c, b^	25 (30.86%)					41 (18.89%)				
Substance use										
Alcohol consumption frequency^d^		1.58	1.00	1.11	3.00		1.68	2.00	1.11	3.00
6+ drinks on one occasion frequency^d^		1.16	1.00	1.15	3.00		1.35	1.00	1.17	3.00
Non-beverage alcohol use frequency^d^		0.31	0.00	0.92	4.00		0.25	0.00	0.72	4.00
Drug use frequency		1.57	2.00	1.27	3.00		1.49	1.00	1.23	3.00
More than one type of drug at the same time frequency^d^		0.54	0.00	1.00	3.00		0.53	0.00	0.94	3.00
Alcohol and drug use at the same time frequency^d^		0.79	0.00	1.02	3.00		0.83	0.00	1.02	3.00
Addiction										
Diagnosed addiction in last year^c^	26 (32.10%)					50 (23.04%)				
Diagnosed addiction previous to last year^c^	18 (22.22%)					56 (25.81%)				
Undiagnosed addiction in last year^c^	11 (13.58%)					40 (18.43%)				
Undiagnosed addiction previous to last year^c^	9 (11.11%)					27 (12.44%)				
Adverse childhood experiences										
ACE score^d, b^		5.02	5.00	2.72	10.00		4.19	4.00	2.97	10.00
Mental health										
Suicidal thoughts in last year^c^	29 (35.8%)					62 (28.57%)				
Suicide attempt in last year^c^	9 (11.11%)					12 (5.53%)				
Suicidal thoughts in lifetime^c, a^	60 (74.07%)					125 (57.60%)				
Suicide attempt in lifetime^c, a^	43 (53.09%)					73 (33.64%)				
Mental health diagnosis^c, a^	51 (62.96%)					80 (36.87%)				
# of diagnosed mental health issues^d, a^		1.85	1.00	1.99	8.00		0.76	0.00	1.29	7.00

Frequencies with percentages provided in parentheses

^a^Significant at the 0.01 level

^b^Significant at the 0.05 level

^c^Independent samples t-test

^d.^ Pearson’s Correlation Coefficient

Table 2 shows that suicide attempts in women’s lifetime and psychiatric hospital stays emerge statistically significant when controlling for age, Indigenous identity, number of times that participants were homeless, and participants’ ACE scores. Women were found to be 1.82 times more likely to have attempted suicide in their lifetime [*p* = 0.04] and twice as likely to report having a psychiatric hospital stay [*p* = 0.03] in model 2 of each respective regression. When factoring in mental illness diagnoses, gender is no longer statistically significant. This suggests that women are at greater risk of staying in a psychiatric hospital and experiencing a suicide attempt not solely due to being female, but because of the likelihood that they will receive a mental illness diagnosis.

**Table 2:** Logistic Regressions

	Suicide Attempt				Psychiatric Stay			
	Unadj.	Model 1	Model 2	Model 3	Unadj.	Model 1	Model 2	Model 3
Female	2.23^a^ (1.33–3.75)	1.82^a^ (1.04–3.16)	1.80^a^ (1.02–3.18)	1.52 (0.85–2.73)	1.92^a^ (1.07–3.43)	1.94^a^ (1.05–3.56)	1.95^a^ (1.05–3.63)	1.45 (0.75–2.79)
Age	0.95^a^ (0.93–0.97)	0.96^a^ (0.94–0.98)	0.97^a^ (0.95–0.99)	0.98 (0.96–1.00)	0.98 (0.96–1.00)	0.98 (0.96–1.01)	0.99 (0.97–1.02)	1.01 (0.98–1.03)
Indigenous identity	2.06^a^ (1.25–3.41)	1.34 (0.77–2.33)	0.96 (0.53–1.72)	1.06 (0.58–1.94)	0.68 (0.36–1.28)	0.50^a^ (0.26–0.99)	0.37^a^ (0.18–0.76)	0.42^a^ (0.20–0.88)
Times homeless	1.22^a^ (1.08–1.38)	1.16^a^ (1.02–1.32)	1.12 (0.98–1.28)	1.10 (0.96–1.26)	1.05 (0.91–1.21)	1.06 (0.92–1.23)	1.02 (0.88–1.19)	0.98 (0.84–1.15)
ACE score	1.31^a^ (1.20–1.43)		1.24^a^ (1.12–1.37)	1.21^a^ (1.09–1.34)	1.16^a^ (1.05–1.28)		1.20^a^ (1.07–1.34)	1.14^a^ (1.01–1.28)
Mental illness diagnosis	4.12^a^ (2.51–6.75)			2.59^a^ (1.50–4.47)	6.46^a^ (3.42–12.20)			5.64^a^ (2.82–11.26)

Odds ratios with 95% confidence intervals provided in parentheses

^a^Significant at the 0.05 level

## Discussion

Our results indicate that a greater proportion of women in our sample experienced suicidal ideation and suicide attempts, childhood trauma, and stays in psychiatric hospitals when compared to male respondents. However, women did not have significantly different rates of substance use from their male counterparts. The literature suggests mental health diagnoses are more prevalent in women than men and mental illness is a significant contributor to women’s homelessness [32, 33]. This is mirrored in the results where mental illness diagnoses acted as a confounding variable, thus suggesting that women are more likely to have a mental illness diagnosis. Being a woman is not statistically significant with the inclusion of a mental illness diagnosis, likely due to the higher prevalence of mental illness among women in our sample (approximately double). In other words, being a woman is not a risk to the outcome, but is highly correlated with other risk factors. Women, therefore, are much more likely to exhibit these health risks than males, implying that front-line service delivery needs respond appropriately.

Given the significance of mental illness and gender, the impact of traumatic experiences need to be considered and factored into the way we support and work with women who are experiencing homelessness.

Experiences of homelessness are gendered and females must be considered independently from their male counterparts. While it is important to consider how women’s experiences of mental illness shapes their experiences of homelessness, policy makers and service providers must also recognize the ways in which gender shapes an individual’s experiences of suicide and psychiatric stays. Women’s higher ACE scores are indicative of both the gendered vulnerability to violence and the prevalence of trauma that women experience in their childhood [19–21]. Ensuring that services are gendered challenges traditional approaches, including many Housing First programs that are gender-neutral. Gender-neutrality is the belief that men and women are the same and therefore should be treated the same [34]. A neutral approach leads to interventions without acknowledgment that there are unique vulnerabilities and experiences associated with gender [35]. This emphasizes the importance of adapting or enhancing Housing First programs to ensure women receive trauma-informed therapeutic supports. For example, organizations that primarily support women could receive funding to implement housing programs. Alternatively, partnerships between existing Housing First programs and women-centred organizations could be facilitated to ensure that staff can provide counselling and/or referrals to trauma-informed counselling services.

As our study demonstrates that women’s experiences of trauma and homelessness are closely interconnected, collaboration must also be facilitated between the trauma/violence and homeless-serving sectors [15]. This would help ensure that Housing First interventions can address the safety needs of women who are vulnerable to previous abusers who may attempt continued exploitation once women are housed.

## Conclusion

This research focuses on female homelessness in order to more closely examine gendered experiences of housing insecurity and plan for both responsive and protective interventions to meet their unique needs. Women’s experiences of homelessness are largely influenced by high rates of adverse childhood experiences and mental health diagnoses. This research points to the need for holistic care that addresses the social determinants of health and provides gender-specific and trauma-informed care.

Intersectoral collaboration and funding specifically for women-centred programming is necessary to bridge gaps between public systems and ensure that interventions including Housing First programs are enhanced to protect women’s safety and provide therapeutic supports to deal with trauma. Future research should focus on ways to engage women who are rough sleeping and/or are staying in unsafe or temporary housing to facilitate access to Housing First programs. Future research should also assess the effectiveness of gendered and trauma-informed Housing First programs in improving women’s health and wellbeing.

## Supplementary information

**Additional file 1: **the adapted *Perceived Need for Care Questionnaire*. This survey was created for a study in Edmonton, Alberta but was adapted to the local context of the research site and a copy was added to the supplementary files section.

## Data Availability

Data may be available by contacting the corresponding author. However, any analysis of the data would need separate ethics approval from corresponding institution and partnership with the original research team.

## Abbreviation

ACE: Adverse childhood experiences
