# Supplementary material for: Recognizing and responding to women experiencing homelessness with gendered and trauma-informed care
Source: BMC Public Health. 2020 Mar 26;20:397. doi: 10.1186/s12889-020-8353-1 (PMC7099814; doi:10.1186/s12889-020-8353-1)
Supplement: Supplementary file 1 — Additional file 1: the adapted Perceived Need for Care Questionnaire. This survey was created for a study in Edmonton, Alberta but was adapted to the local context of the research site and a copy was added to the supplementary files section. [file 12889_2020_8353_MOESM1_ESM.docx]

**Perceived Need for Health Services amongst Persons Experiencing Homelessness Who Use Drugs and Alcohol**

**Eligibility Criteria:**

□ 16 years or older

□ Been homeless for more than six months

**OR**

□ Have had at least four episodes of homelessness within the last two years.

□ Has provided informed consent

**Participant will receive $25 compensation for their time.**

**Interview date: __ __/__ __/__ __ __ __**

DD MM YEAR

**Survey code: ___________________________________**

*This is to ensure we do not get duplicates.*

**Interview location**

- Calgary Drop In Centre
- Alpha House
- Inn From The Cold
- Outside
- Other____________________

**Interviewer initials:**_____________

**Interview start time**: ____________ AM or PM (*please circle*)

**Is the information collected in the interview significantly distorted by the participant’s misrepresentation?**

□ No

□ Yes

**Is the information collected in the interview significantly distorted by the participant’s inability to understand?**

□ No

□ Yes

**Interview date: __ __/__ __/__ __ __ __**

DD MM YEAR

**Name (Will be anonymized later): ___________________________________**

**Interview location**

□ Calgary Drop In Centre

□ Alpha House

□ Mustard Seed

□ Inn From The Cold

□ Other

**Interviewer initials:**_____________

**Interview start time**: ____________ AM or PM (*please circle*)

**Is the information collected in the interview significantly distorted by the participant’s misrepresentation?**

□ No

□ Yes

**Is the information collected in the interview significantly distorted by the participant’s inability to understand?**

□ No

□ Yes


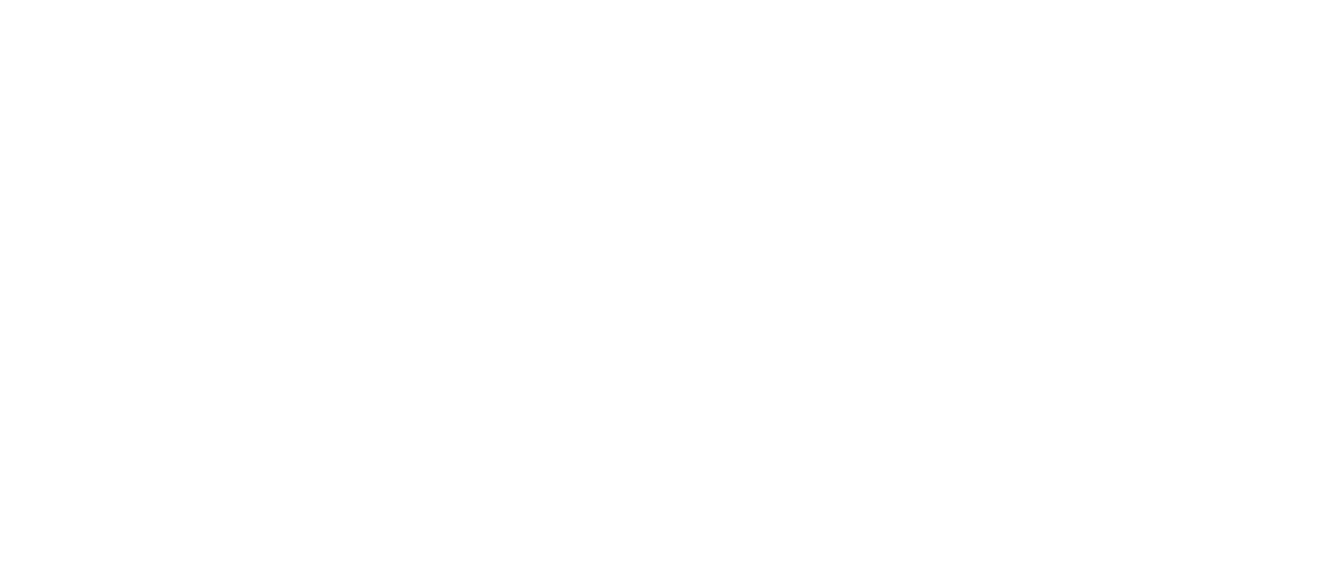


***Thank you for coming in today and contributing to this study. As we go through the interview together, please keep in mind that there are no wrong answers.***

***It’s very important that you answer as honestly as you can. We rely on your information to help create positive change for people.***

***We realize some of these questions are sensitive. If you do not want to answer***

***a question, just let me know and we will move on. It is better for you to refuse to answer a question than to give a false answer. False answers affect the quality of our data and limit our ability to advocate for positive change.***

***We take your privacy very seriously. All the information that you provide will only be kept between you and me. We never report any individual information. If there are any questions you don’t understand, please stop me and ask for clarification. The interview takes about half an hour. If you need a break, let me know and we can stop for a short rest before we finish the interview.***


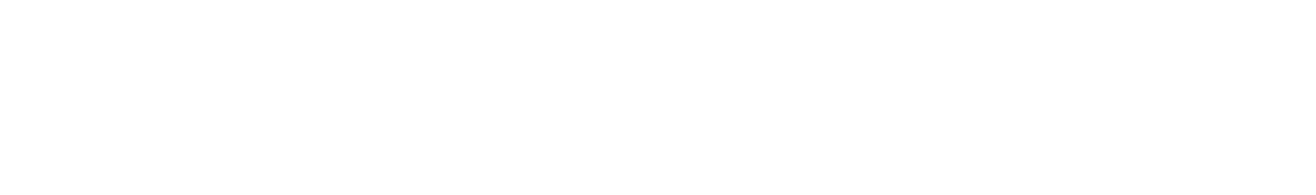


***In this first section, I’m going to ask you some questions about your personal history, your drug use, and your health. Some of these questions are very personal. Please remember that the answers you give are totally confidential. We are asking everyone who participates the same questions.***

**PART 1. SOCIO-DEMOGRAPHICS**

**1. Over the course of your entire life, how long have you been homeless?**

- 6-11 months
- 1 year
- 2-3 years
- 4-5 years
- 6-7 years
- 8-9 years
- 10-11 years
- More than 11 years

**a.) How many times have you been homeless in your life?**

- Once
- Twice
- Three times
- Four times
- Five times
- More than five times

**2. How old are you?_________years**

**3. What is your gender?**

□ Male

□ Female

□ Transgendered

□ Other (specify):__________

□ Don’t know

□ Refused

**4. What ethnic group or family background do you most closely identify with?**

(Do NOT read out list.)

□ Caucasian/White

□ South Asian (e.g. Indian, Pakistani)

□ Chinese

□ Other Asian

□ Latin American

□ Middle Eastern

□ Black African

□ Black Caribbean

□ First Nations/Aboriginal

□ Inuit

□ Metis

□ Other (Specify):_____________

□ Don’t know

□ Refused

**a). [Do Not Read] The participant self-identifies as being of First**

**Nations, Aboriginal, Inuit or Metis ancestry:**

□ Yes (Go to **‘b’**)

□ No (Go to **Q.5**)


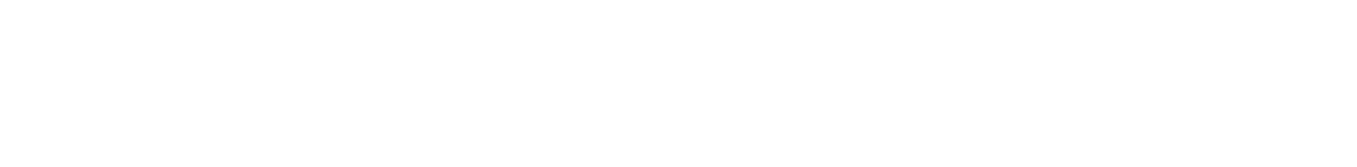


**Next I am going to ask you about you and your family’s experience with residential schools. We know that talking about residential school may be sensitive and potentially upsetting for some people. I just want to remind you that anything you say to us is confidential and you have the right to refuse to answer any questions.**

**b). Did anyone in your family ever attend a residential school?**

□ Yes (go to **‘c”)**

□ No (go to **Q.d**)

□ Don’t know

□ Refused

**c). [IF YES] Who?** [**Do not read list. Check ALL that apply**]

□ Mother

□ Father

□ Sibling(s)

□ Children

□ Grandmother(s)

□ Grandfather(s)

□ Aunt(s)

□ Uncle(s)

□ Cousin(s)

□ Other:____________________

**d). Did you ever attend a residential school?**

□ Yes (Go to **‘e’)**

□ No (go to **Q.5**)

□ Don’t know

□ Refused

**e). [IF YES] For how many years approximately? ______________**

**5. Have you ever been in foster care or been removed from your family for any reason?**

- - Yes
  - No
  - Don’t know
  - Refused

**6. What is your marital status?**

- Married
- Divorced
- Widowed
- Single
- Common law
- Other_______________________

**7. Do you have children?**

- - Yes (**Go to ‘a’)**
  - No (**Go to ‘b’)**
  - Don’t know
  - Refused

**a). If yes, are they in your care?**

- - Yes
  - No
  - Don’t know
  - Refused

**b). If not in your care, where are they?**

- Grown up
- With family
- With friends
- In foster care/child welfare
- Other_____________________
- Don’t know
- Refused to answer

**8. How long have you lived in Calgary**? __________

- Less than 1 year
- 1-5 years
- 6-10 years
- More than 10 years
- My whole life (go to **Q.8**)
- Don’t know
- Refused

a. **Where did you live just before you came to Calgary?**

______________

□ Don’t know

□ Refused

**9. In the past 6 months, what types of places have you slept in?** (**Read out the list and check ALL that apply**).

- - Own apartment/house
  - Hotel/furnished room/boarding house
  - Transition housing
  - Shelter/hostel
  - Friend’s place
  - Family member’s place
  - Outside/Camps
  - Working out of town (rigs/work camp)
  - Reserve or settlement
  - Couch surfing
  - Detox
  - Jail/prison
  - Hospital
  - Vehicle
  - Don’t sleep (walk all night)
  - Don’t know
  - Refused

**PART 2: ACE**

**Now, I am going to ask you some very personal questions about your early childhood. A lot of research now shows that Adverse Childhood Experiences (ACE) contribute to problems later in life such as obesity, heart disease, mental health problems, and different types of addiction. Your responses will help us understand how child maltreatment and family dysfunction are related to homelessness and health outcomes. While the questions often say “parent” and “household”, we’d like to know if any of these experiences happened to you in any place you lived in while you were growing up. This could include a relative’s home, foster home, group home, residential school, or halfway house. We know that talking about Adverse Childhood Experiences may be sensitive and potentially upsetting for some people. I just want to remind you that anything you say to us is confidential and you have the right to refuse to answer any questions.**

**Note: You may wish to do this section privately.**

***Prior to your 18^th^ birthday:***

10. Did a parent or other adult in the household **often or very often**…

Swear at you, insult you, put you down, or humiliate you?

**or**

Act in a way that made you afraid that you might be physically hurt?

Yes No If yes enter 1 ________

11. Did a parent or other adult in the household **often or very often**…

Push, grab, slap, or throw something at you?

**or**

**Ever** hit you so hard that you had marks or were injured?

Yes No If yes enter 1 ________

12. Did an adult or person at least 5 years older than you **ever**…

Touch or fondle you or have you touch their body in a sexual way?

**or**

Attempt or actually have oral, anal, or vaginal intercourse with you?

Yes No If yes enter 1 ________

13. Did you **often or very often** feel that …

No one in your family loved you or thought you were important or special?

**or**

Your family didn’t look out for each other, feel close to each other, or support each other?

Yes No If yes enter 1 ________

14. Did you **often or very often** feel that …

You didn’t have enough to eat, had to wear dirty clothes, and had no one to protect you?

**or**

Your parents were too drunk or high to take care of you or take you to the doctor if you needed it?

Yes No If yes enter 1 ________

15. Was a biological parent **ever** lost to you through divorced, abandonment, or other reason?

Yes No If yes enter 1 ________

16. Was your mother or stepmother:

**Often or very often** pushed, grabbed, slapped, or had something thrown at her?

**or**

**Sometimes, often, or very often** kicked, bitten, hit with a fist, or hit with something hard?

**or**

**Ever** repeatedly hit over at least a few minutes or threatened with a gun or knife?

Yes No If yes enter 1 ________

17. Did you live with anyone who was a problem drinker or alcoholic or who used street drugs?

Yes No If yes enter 1 ________

18. Was a household member depressed or mentally ill or did a household member attempt suicide?

Yes No If yes enter 1 ________

19. Did a household member go to prison?

Yes No If yes enter 1 ________

**PART 3. DRUG USE, RISK BEHAVIOURS, AND EXPERIENCE OF HARM**

**The next few questions are about your tobacco and alcohol use, and any drugs you might use. This includes illicit drugs and prescription drugs that you take without a prescription from a doctor or for nonmedical reasons.**

**20. Do you use tobacco products such as cigarettes, cigars, or chewing tobacco?**

□ Yes

□ No

□ Don’t know

□ Refused

**21. How often do you have a drink containing alcohol? Alcohol means beer, wine, or hard liquor?**

□ Never (**Go to Q.22)**

□ Monthly or less

□ Weekly

□ Daily or almost daily

□ Don’t know

□ Refused

**22. How often do you have six or more drinks on one occasion? One drink means 12 oz beer, 5 ounces of wine, or 1.5 oz of hard liquor.**

□ Never

□ Monthly or less

□ Weekly

□ Daily or almost daily

□ Don’t know

□ Refused

**23. Have you ever used non-beverage alcohol (cooking wine/rubbing alcohol/mouthwash/cologne) when you couldn’t access regular alcohol?**

□ Yes

□ No (Go to **Q.24)**

□ Don’t know

□ Refused

**24. If yes, how often do you use non-beverage alcohol?**

□ Yearly or less

□ Monthly or less

□ Weekly

□ Daily or almost daily

□ Don’t know

□ Refused

**25. Over the past year, have you felt that your longing for alcohol was so strong that you could not resist it?**

□ Never

□ Monthly or less

□ Weekly

□ Daily or almost daily

□ Don’t know

□ Refused

**26. How often over the past year have you had guilt feelings or a bad conscience because you used alcohol?**

□ Never

□ Less often than once a month

□ Every month

□ Every week

□ Daily or almost every day

□ Don’t know

□ Refused

**27. Have you or anyone else been hurt (mentally or physically) because you used alcohol?**

□ No

□ Yes, but not over the past year

□ Yes, over the past year

□ Don’t know

□ Refused

**28. How often do you use drugs other than alcohol? Drugs means illicit drugs and prescription drugs that you take without a prescription from a doctor or for nonmedical reasons.**

□ Never

□ Monthly or less

□ Weekly

□ Daily or almost daily

□ Don’t know

□ Refused

**29. Do you use more than one type of drug at the same time?**

□ Never

□ Monthly or less

□ Weekly

□ Daily or almost daily

□ Don’t know

□ Refused

**30. How often do you use alcohol and drugs at the same time?**

□ Never

□ Monthly or less

□ Weekly

□ Daily or almost daily

□ Don’t know

□ Refused

**31. Which substances have you taken in the last six months? (read and check all that apply)**

- Alcohol
- Non-beverage alcohol (cooking wine/rubbing alcohol/mouthwash)
- Marijuana
- Crack
- Amphetamine
- Methamphetamine
- OxyContin
- Cocaine
- Heroin
- Opium
- Hashish
- MDMA
- Fentanyl
- Inhalants (solvents)
- Other ______________

**32. Which substances do you take most often? Please rank 1^st^, 2^nd^, 3^rd^ etc…)**

- Alcohol
- Non-beverage alcohol (cooking wine/rubbing alcohol/mouthwash)
- Marijuana
- Crack
- Amphetamine
- Methamphetamine
- OxyContin
- Cocaine
- Heroin
- Opium
- Hashish
- MDMA
- Fentanyl
- Inhalants (solvents)

Other ______________

**33. Over the past year, have you felt that your longing for drugs was so strong that you could not resist it?**

□ Never

□ Monthly or less

□ Weekly

□ Daily or almost daily

□ Don’t know

□ Refused

**34. How often over the past year have you had guilt feelings or a bad conscience because you used drugs?**

□ Never

□ Less often than once a month

□ Every month

□ Every week

□ Daily or almost every day

□ Don’t know

□ Refused

**35. Have you or anyone else been hurt (mentally or physically) because you used drugs?**

□ No

□ Yes, but not over the past year

□ Yes, over the past year

□ Don’t know

□ Refused

**36. Have you ever become physically violent when you were under the influence of alcohol or drugs?**

□ No

□ Yes, but not in the last year

□ Yes, during the last year

□ Don’t know

□ Refused

**37. Have you ever suffered a health problem because of your drinking or drug use?**

□ No (go to **26**)

□ Yes, but not in the last year

□ Yes, during the last year

□ Don’t know

□ Refused

**a. If yes, what was the health problem? _____________**

□ Don’t know

□ Refused

**38. Have you ever had legal difficulties—for example, been prosecuted, been arrested by the police or gotten a big fine—as a result of drinking or drug use?**

□ No

□ Yes, but not in the last year

□ Yes, during the last year

□ Don’t know

□ Refused

**PART 4: Health Status**

**Now I am going to ask you a few questions about your health status – that means your physical and mental health, including addictions, and the problems and stresses that you might face in your day to day life.**

**39. In the last 12 months have you been physically attacked or assaulted?**

□ Yes **(Go to Q. 40)**

□ No (go to **Q. 41)**

□ Don’t know

□ Refused

**40. If yes, how many times?**

- Once
- 2-3
- 4-5
- More than 5

**41. Have you ever hit your head so hard you lost consciousness?**

□ Yes (**go to Q. 42)**

□ No (go to **Q.43**)

□ Don’t know

□ Refused

**42. If yes, how many times?**

- Once
- 2-3
- 4-5
- More than 5

**43. Have you ever been diagnosed with any of the following disabilities? (read and check all that apply)**

- FASD
- Autism
- ADD/ADHD
- Learning disability
- Other ______________________
- Don’t know
- Refused

**44. If no, do you think you have a disability that has not been diagnosed?**

- Yes (**go to Q. 45)**
- No (**go to Q. 46)**
- Don’t know
- Refused to answer

**45. If yes, which one?**

- FASD
- Autism
- ADD/ADHD
- Learning disability
- Other ______________________

**46. In the last 12 months have you done anything to harm yourself? (read and check as many as apply)**

- Cutting
- Burning
- Scratching
- Hitting your body
- Hair pulling
- Other ________________

**47. In the last 12 months have you thought about committing suicide?**

- Yes
- No
- Don’t know
- Refused

**48. In the last 12 months have you attempted suicide?**

- Yes
- No
- Don’t know
- Refused

**49. In your lifetime have you done anything to harm yourself? (read and check as many as apply)**

- Cutting
- Burning
- Scratching
- Hitting your body
- Hair pulling
- Other ________________

**50. In your lifetime have you thought about committing suicide?**

- Yes
- No
- Don’t know
- Refused

**50. In your lifetime have you attempted suicide?**

- Yes
- No
- Don’t know
- Refused

**51. Has a health professional ever diagnosed you with a health condition?**

- Yes
- No (go to Q.X)
- Don’t know
- Refused to answer

**52. If yes, what is it (read and check all that apply)**

- AIDS/HIV
- Hepatitis A
- Hepatitis B
- Hepatitis C
- Sexually transmitted disease
- Cancer
- Diabetes
- High blood pressure
- Stroke
- Heart attack
- Arthritis
- Asthma
- COPD (chronic obstructive pulmonary disease)
- Emphysema
- Bronchitis
- Brain/head injury
- Chronic pain
- Frostbite
- Mobility issues (problems walking or doing other basic tasks)
- Skin and foot problems (scabies, lice, impetigo, calluses, corns, tine pedis)
- Dental problems (dental surgery, root canals, gum disease)
- Other____________________________

**53. Do you experience chronic or recurring health conditions or ongoing challenges related to your physical health because of homelessness** (e.g. chronic pain, frostbite, skin and foot problems, injuries/wounds, dental problems etc.)?

- Yes
- No
- Don’t know
- Refused to answer

**a). If yes, please list them _________________________________________**

**54. Has a health professional ever told you that you have a mental health condition?**

□ Yes, in the past 12 months

□ Yes, but not in the past 12 months

□ *Yes (only if unable to specify time period*)

□ No

□ Don’t know

□ Refused

**a). If yes, which mental health condition? (read and check all that apply)**

- Anxiety
- Depression
- Bi-polar
- Schizophrenia
- Post-traumatic stress disorder (PTSD)
- Psychotic disorder
- Other _________________

**55. Do you think you have ever had a mental health condition that has not been diagnosed by a professional?**

□ Yes, in the past 12 months

□ Yes, but not in the past 12 months

□ Yes (*only if unable to specify time period*)

□ No

□ Don’t know

□ Refused

**56. If yes, which mental health condition? (read and check all that apply)**

- Anxiety
- Depression
- Bi-polar
- Schizophrenia
- Post-traumatic stress disorder (PTSD)
- Psychotic disorder
- Other__________________

**57. Has a health professional ever told you that you have an addiction?** *(By addiction I mean misuse of things like alcohol, street drugs, or prescription medications to get high, or engaging in behaviours like gambling, or sex in a way that creates problems in life*)?

□ Yes, in the past 12 months

□ Yes, but not in the past 12 months

□ *Yes (only if unable to specify time period*)

□ No

□ Don’t know

□ Refused

**58. If yes, what addiction? (check as many as apply)**

- Alcohol
- Illicit drugs
- Gambling
- Sex
- Food
- Other ______________

**59. Do you think you have ever had an addiction that has not been diagnosed by a professional?** *(By addiction I mean misuse of things like alcohol, street drugs, or prescription medications to get high, or engaging in behaviours like gambling, or sex in a way that creates problems in life*)?

□ Yes, in the past 12 months

□ Yes, but not in the past 12 months

□ Yes (*only if unable to specify time period*)

□ No□ Don’t know

□ Refused

**60. If yes, what addiction problem? (check as many as apply)**

- Alcohol
- Illicit drugs
- Gambling
- Sex
- Food
- Other ______________

**61. We’d like to get a sense of what sorts of things in your life you find stressful right now. Please rate each source of stress on a scale of 1 to 5, with 1 being no stress at all, 2 being slight stress, 3 being an average amount of stress, 4 being high stress, and 5 being very high stress.**

a). Money ___________

b)**.** Job or employment________________

c). Physical health condition______________

d). Mental health condition__________________

e). Addiction_________________

f). Housing_____________

g). Legal problems______________

h). Parents/siblings__________________

i). Relationship (partner/boyfriend/girlfriend/spouse) __________

j). Children__________________

**62. What would you say your overall level of stress has been in the past 6 months?**

- No stress
- Slight stress
- Average stress
- High stress
- Very high stress

**63. How do you deal with the stress in your life right now? (read and check all that apply)**

- Using alcohol or drugs/getting high
- Becoming aggressive or violent
- Avoiding contact with people
- Seeking contact with people
- Asking for help
- Other __________________________

**PART 4: Accessing Services**

**Now I am going to ask you a few questions about how you access services and supports such as health care, housing, employment, or other services, and family, friends and other relationships that might help you in your day to day life.**

**64. In the past 12 months, have you received information about treatments or available services because of problems with your physical health? (Read out list. Check ONE).**

□ Yes, in the past 12 months (go to ‘**a & b**’)

□ No, but I think I needed this kind of help in the past 12 months (**go to ‘c’)**

□ No, I did not need this kind of help in the past 12 months (**go to Q. 65**).

□ Don’t know

□ Refused

**a). Where or from whom did you get this information?**

- Shelter
- Detox
- Outreach worker
- Emergency medical technician (EMT/ambulance service)
- Hospital emergency room
- Hospital (other than ER)
- Health clinic (non-hospital)
- Police/peace/bylaw officer
- Community service agency not otherwise specified
- Housing worker
- Don’t know
- Refused to answer

**b. Do you think you got as much information as you needed?**

□ Yes (**go to Q. 65)**

□ No (go to ‘**c**’)

□ Don’t know

□ Refused

**c. Please indicate if each of the following reasons stopped you from getting any or enough help in the past 12 months (Check all that apply)**

□ I preferred to manage myself

□ I didn’t know where to get help

□ I was afraid to ask for help or what others would think of me

□ I couldn’t afford the money

- I had no transportation
- I didn’t qualify for help (because of housing/legal/addiction/mental health status)

□ I asked but didn’t get help

□ I didn’t think anything would help/nothing will help me

□ I don’t want to get help at this time

□ Wait list too long/no spaces available

□ I was only allowed a limited amount of [information]

□ Other:________________

□ Don’t know

□ Refused

**65. In the past 12 months, have you received treatments or medication (e.g., tablets to help you with these problems) because of problems with your physical health?** *(e.g. insulin, anti-hypertensives, chemotherapy, antibiotics, antiretrovirals, wheelchair/occupational health need, etc.)* **(Read out list. Check ONE).**

□ Yes, in the past 12 months (go to ‘**a & b**’)

□ No, but I think I needed this kind of help in the past 12 months **(go to ‘c’)**

□ No, I did not need this kind of help in the past 12 months (**go to Q. 66)**

□ Don’t know

□ Refused

**a). Where or from whom did you access the treatment?**

- Shelter
- Detox
- Outreach worker
- Emergency medical technician (EMT/ambulance service)
- Hospital emergency room
- Hospital (other than ER)
- Health clinic (non-hospital)
- Police/peace/bylaw officer
- Community service agency not otherwise specified
- Housing worker
- Don’t know
- Refused to answer

**b). Do you think you got as much treatment as you needed?**

□ Yes (**go to Q. 66)**

□ No (**go to ‘c’)**

□ Don’t know

□ Refused

**c). Please indicate if each of the following reasons stopped you from getting any or enough treatments in the past 12 months (Check all that apply)**

□ I preferred to manage myself

□ I didn’t know where to get help

□ I was afraid to ask for help or what others would think of me

□ I couldn’t afford the money

- I had no transportation
- I didn’t qualify for help (because of housing/legal/addiction/mental health status)

□ I asked but didn’t get help

□ I didn’t think anything would help/nothing will help me

□ I don’t want to get help at this time

□ Wait list too long/no spaces available

□ I was only allowed a limited amount of [information]

□ Other:________________

□ Don’t know

□ Refused

**66. In the past 12 months, have you received information about treatments or available services because of problems with your emotions, mental health, or use of alcohol or drugs? (Read out list. Check ONE).**

□ Yes, in the past 12 months (go to ‘**a & b**’)

□ No, but I think I needed this kind of help in the past 12 months (**go to ‘c’)**

□ No, I did not need this kind of help in the past 12 months **(go to Q. 67)**

□ Don’t know

□ Refused

**a). Where or from whom did you get this information?**

- Shelter
- Detox
- Outreach worker
- Emergency medical technician (EMT/ambulance service)
- Hospital emergency room
- Hospital (other than ER)
- Health clinic (non-hospital)
- Police/peace/bylaw officer
- Community service agency not otherwise specified
- Housing worker
- Don’t know
- Refused to answer

**b). Do you think you got as much information as you needed?**

□ Yes (**go to Q. 67)**

□ No (go to ‘**c**’)

□ Don’t know

□ Refused

**c). Please indicate if each of the following reasons stopped you from getting any or enough help in the past 12 months (Check all that apply)**

□ I preferred to manage myself

□ I didn’t know where to get help

□ I was afraid to ask for help or what others would think of me

□ I couldn’t afford the money

- I had no transportation
- I didn’t qualify for help (because of housing/legal/addiction/mental health status)

□ I asked but didn’t get help

□ I didn’t think anything would help/nothing will help me

□ I don’t want to get help at this time

□ Wait list too long/no spaces available

□ I was only allowed a limited amount of [information]

□ Other:________________

□ Don’t know

□ Refused

**67. In the past 12 months, have you received medication (or tablets to help you with these problems) because of problems with your emotions, mental health, or use of alcohol or drugs?** *(e.g. methadone, antidepressants, etc.)* **(Read out list. Check ONE).**

□ Yes, in the past 12 months (go to ‘**a & b**’)

□ No, but I think I needed this kind of help in the past 12 months (**go to ‘c’)**

□ No, I did not need this kind of help in the past 12 months **(go Q. 68**)

□ Don’t know

□ Refused

**a). Where or from whom did you get this medication?**

- Shelter
- Detox
- Outreach worker
- Emergency medical technician (EMT/ambulance service)
- Hospital emergency room
- Hospital (other than ER)
- Health clinic (non-hospital)
- Police/peace/bylaw officer
- Community service agency not otherwise specified
- Housing worker
- Don’t know
- Refused to answer

**b). Do you think you got as much medication as you needed?**

□ Yes (**go to Q. 68)**

□ No (**go to ‘c’)**

□ Don’t know

□ Refused

**c). Please indicate if each of the following reasons stopped you from getting any or enough help in the past 12 months (Check all that apply)**

□ I preferred to manage myself

□ I didn’t think anything would help

□ I didn’t know where to get help

□ I was afraid to ask for help or what others would think of me

□ I couldn’t afford the money

- I had no transportation
- I didn’t qualify for help (because of housing/legal/addiction/mental health status)

□ I asked but didn’t get help

□ I don’t want to get help at this time

□ Wait list too long/no spaces available

□ I was only allowed a limited amount of [medication]

□ Other: ________________

□ Don’t know

□ Refused

**68. In the past 12 months, have you received hospital care (overnight or longer) because of problems with your physical or mental health, or use of alcohol or drugs?** (E.g. treating an infection or abscess, overdose, psychosis, etc.) **(Read out list. Check ONE).**

□ Yes, in the past 12 months (go to ‘**a**)

□ No, but I think I needed this kind of help in the past 12 months (**go to ‘b’)**

□ No, I did not need this kind of help in the past 12 months (**go to Q. 69)**

□ Don’t know

□ Refused

**a). Do you think you got as much hospital care as you needed?**

□ Yes (go to **Q.61**)

□ No (go to ‘**b**’)

□ Don’t know

□ Refused

**b). Please indicate if each of the following reasons stopped you from getting any or enough help in the past 12 months (Check all that apply)**

□ I preferred to manage myself

□ I didn’t think anything would help

□ I didn’t know where to get help

□ I was afraid to ask for help or what others would think of me

□ I couldn’t afford the money

- I had no transportation
- I didn’t qualify for help (because of housing/legal/addiction/mental health status)

□ I asked but didn’t get help

□ I don’t want to get help at this time

□ Wait list too long/no spaces available

□ I was only allowed a limited [amount of time in hospital]

□ Other: ________________

□ Don’t know

□ Refused

**69. In the past 12 months, have you received detox because of problems with your emotions, mental health, or use of alcohol or drugs?** (e.g. addiction, overdose, psychosis, etc.) **(Read out list. Check ONE).**

□ Yes, in the past 12 months (go to ‘**a**’)

□ No, but I think I needed this kind of help in the past 12 months (go to ‘**b**’)

□ No, I did not need this kind of help in the past 12 months (**go to Q. 70**)

□ Don’t know

□ Refused

**a. Do you think you got as much detox or treatment care as you needed?**

□ Yes (go to **Q.70**)

□ No (go to ‘**b**’)

□ Don’t know

□ Refused

**b. Please indicate if each of the following reasons stopped you from getting any or enough help in the past 12 months (Check all that apply)**

□ I preferred to manage myself

□ I didn’t think anything would help

□ I didn’t know where to get help

□ I was afraid to ask for help or what others would think of me

□ I couldn’t afford the money

- I had no transportation
- I didn’t qualify for help (because of housing/legal/addiction/mental health status)

□ I asked but didn’t get help

□ I don’t want to get help at this time

□ Wait list too long/no spaces available

□ I was only allowed a limited [amount of time in hospital]

□ Other: ________________

□ Don’t know

□ Refused

**70. In the past 12 months, have you received residential treatment (overnight or longer) because of problems with your emotions, mental health, or use of alcohol or drugs?** (e.g. addiction, overdose, psychosis, etc.) **(Read out list. Check ONE).**

□ Yes, in the past 12 months (go to ‘**a**’)

□ No, but I think I needed this kind of help in the past 12 months (go to ‘**b**’)

□ No, I did not need this kind of help in the past 12 months (**go to Q. 71)**

□ Don’t know

□ Refused

**a. Do you think you got as much treatment as you needed?**

□ Yes (go to **Q.71**)

□ No (go to ‘**b**’)

□ Don’t know

□ Refused

**b. Please indicate if each of the following reasons stopped you from getting any or enough help in the past 12 months (Check all that apply)**

□ I preferred to manage myself

□ I didn’t think anything would help

□ I didn’t know where to get help

□ I was afraid to ask for help or what others would think of me

□ I couldn’t afford the money

- I had no transportation
- I didn’t qualify for help (because of housing/legal/addiction/mental health status)

□ I asked but didn’t get help

□ I don’t want to get help at this time

□ Wait list too long/no spaces available

□ I was only allowed a limited [amount of time in hospital]

□ Other: ________________

□ Don’t know

□ Refused

**71. In the past 12 months, have you received outpatient services [medical tests, day surgery] because of problems with your physical or mental health, or use of alcohol or drugs?** (E.g. treating an infection or abscess, overdose, psychosis, etc.) **(Read out list. Check ONE).**

□ Yes, in the past 12 months (go to ‘**a**’)

□ No, but I think I needed this kind of help in the past 12 months (go to ‘**b**’)

□ No, I did not need this kind of help in the past 12 months (**go to Q. 72)**

□ Don’t know

□ Refused

1. **Where or from whom did you get this service?**

- Hospital emergency room
- Hospital (other than ER)
- Health clinic (non-hospital)
- Community service agency not otherwise specified
- Don’t know
- Refused to answer

**a. Do you think you got as much outpatient care as you needed?**

□ Yes (go to **Q. 72**)

□ No (go to ‘**b**’)

□ Don’t know

□ Refused

**b. Please indicate if each of the following reasons stopped you from getting any or enough help in the past 12 months (Check all that apply)**

□ I preferred to manage myself

□ I didn’t think anything would help

□ I didn’t know where to get help

□ I was afraid to ask for help or what others would think of me

□ I couldn’t afford the money

- I had no transportation
- I didn’t qualify for help (because of housing/legal/addiction/mental health status)

□ I asked but didn’t get help

□ I don’t want to get help at this time

□ Wait list too long/no spaces available

□ I was only allowed a limited [amount of time in hospital]

□ Other: ________________

□ Don’t know

□ Refused

**72. In the past 12 months, have you received counselling (outside of a hospital including any kind of help to talk through your problems) because of problems with your physical or mental health, or use of alcohol or drugs? (Read out list. Check ONE).**

□ Yes, in the past 12 months (go to ‘**a & b**’)

□ No, but I think I needed this kind of help in the past 12 months (go to **‘c’**)

□ No, I did not need this kind of help in the past 12 months (go **to Q. 73**)

□ Don’t know

□ Refused

**a. Do you think you got as much counselling as you needed?**

□ Yes (go to **‘b’)**

□ No (go to ‘**c**’)

□ Don’t know

□ Refused

**b).Where or from whom did you get this counselling?**

- Shelter
- Health clinic (non-hospital)
- Community service agency not otherwise specified
- Don’t know
- Refused to answer

**c). Please indicate if each of the following reasons stopped you from getting any or enough help in the past 12 months (Check all that apply)**

□ I preferred to manage myself

□ I didn’t think anything would help

□ I didn’t know where to get help

□ I was afraid to ask for help or what others would think of me

□ I couldn’t afford the money

- I had no transportation
- I didn’t qualify for help (because of housing/legal/addiction/mental health status)

□ I asked but didn’t get help

□ I don’t want to get help at this time

□ Wait list too long/no spaces available

□ I was only allowed a limited amount of [sessions/appointments]

□ Other: ________________

□ Don’t know

□ Refused

**73. In the past 12 months, have you received social interventions because of homelessness** (E.g. income support, shelter, housing, employment support/ training, legal support, identification)

□ Yes, in the past 12 months (go to ‘**a & b**’)

□ No, but I think I needed this kind of help in the past 12 months (go to ‘**c**’)

□ No, I did not need this kind of help in the past 12 months (go to **Q. 74**)

□ Don’t know

□ Refused

**a. Do you think you got as much social interventions (for housing or money problems) as you needed?**

□ Yes (**go to ‘b’)**

□ No (go to ‘**c**’)

□ Don’t know

□ Refused

**b. What kind of interventions (check as many as apply)**

- income support,
- shelters,
- housing,
- employment
- support training,
- legal support,
- identification,
- other

**c. Please indicate if each of the following reasons stopped you from getting any or enough help in the past 12 months (Check all that apply)**

□ I preferred to manage myself

□ I didn’t think anything would help

□ I didn’t know where to get help

□ I was afraid to ask for help or what others would think of me

□ I couldn’t afford the money

- I had no transportation
- I didn’t qualify for help (because of housing/legal/addiction/mental health status)

□ I asked but didn’t get help

□ I don’t want to get help at this time

□ Wait list too long/no spaces available

□ I was only allowed a limited amount of [social interventions/help]

□ Other: ________________

□ Don’t know

□ Refused

**74. In the past 12 months, have you received access to harm reduction (services [like needle exchange, condoms, crack pipes etc…] to reduce the risk of harm related to using drugs) because of problems with your use of alcohol or drugs? (Read out list. Check ONE).**

□ Yes, in the past 12 months (go to ‘**a**’)

□ No, but I think I needed this kind of help in the past 12 months (go to ‘**b**’)

□ No, I did not need this kind of help in the past 12 months (go to **Q. 75**)

□ Don’t know

□ Refused

**a. Do you think you got as much harm reduction as you needed?**

□ Yes (**go to ‘b’**)

□ No (go to ‘**c**’)

□ Don’t know

□ Refused

**b. Where or from whom did you get this from?**

- Shelter
- Detox
- Outreach worker
- Emergency medical technician (EMT/ambulance service)
- Hospital emergency room
- Hospital (other than ER)
- Health clinic (non-hospital)
- Police/peace/bylaw officer
- Community service agency not otherwise specified
- Housing worker
- Don’t know
- Refused to answer

**c. Please indicate if each of the following reasons stopped you from getting any or enough help in the past 12 months (Check all that apply)**

□ I preferred to manage myself

□ I didn’t think anything would help

□ I didn’t know where to get help

□ I was afraid to ask for help or what others would think of me

□ I couldn’t afford the money

- I had no transportation
- I didn’t qualify for help (because of housing/legal/addiction/mental health status)

□ I asked but didn’t get help

□ I don’t want to get help at this time

□ Wait list too long/no spaces available

□ I was only allowed a limited amount of [harm reduction services/supplies]

□ Other:________________

□ Don’t know

□ Refused

**75. At any time in your life have you been a resident of a psychiatric hospital, ward, facility or center for people with mental illness or disabilities?**

- Yes (go to **a,b,c**)
- No (go to **Q. 76)**
- Don’t know
- Refused

**a). How many times have you been a resident?**

- Once
- 2-3 times
- 4-5 times
- More than 5 times

**b). What is the longest amount of time spent there?**

- Several days
- Several weeks
- Several months
- Several years
- Don’t know
- Refused

**c). Were you ever discharged because the hospital, ward, facility or center was closing?**

- Yes
- No
- Don’t know
- Refused

**76. After you left the hospital, where did you go?**

- Own home
- Someone else’s home
- Another facility
- Jail
- The street

**Now I am going to ask you a few questions about your experiences with health, housing, community, and justice services.**

**77. Overall, how satisfied are you with your health care services?**

- Very satisfied
- Satisfied
- Unsatisfied
- Very unsatisfied
- Don’t know
- Refused to answer

**78. How easy is it to access health care when you need it?**

- Very easy
- Relatively easy
- Difficult
- Very difficult
- Don’t know
- Refused to answer

**79. In your experience are health care practitioners caring and compassionate?**

- Always
- Often
- Sometimes
- Never
- Don’t know
- Refused to answer

**80. Overall, how satisfied are you with your housing services?**

- Very satisfied
- Satisfied
- Unsatisfied
- Very unsatisfied
- Don’t know
- Refused to answer

**81. How easy is it to access housing when you need it?**

- Very easy
- Relatively easy
- Difficult
- Very difficult
- Don’t know
- Refused to answer

**82. In your experience are housing workers caring and compassionate?**

- Always
- Often
- Sometimes
- Never
- Don’t know
- Refused to answer

**83. Has anything happened to you in the last 12 months that required police assistance?**

- Yes (go to ‘**a’**)
- No (go to **Q. 84**)
- Don’t know
- Refused

**a). Did you feel that (check all that apply)**

- Response was prompt
- Response was effective
- There was no response
- Response was ineffective

**84. Has anything happened to you in the last 12 months that required emergency medical assistance?**

- Yes (go to **‘a’**)
- No (go to **Q. 85**)
- Don’t know
- Refused

**a. Did you feel that (check all that apply)**

- Response was prompt
- Response was effective
- There was no response
- Response was ineffective

**85. Do you have access to any social supports, such as friends, family or a church or faith group?**

- Yes (go to ‘**a**’)
- No (go to **Q.86)**
- Don’t know
- Refused to answer

**a). If yes, who are they? (read and check all that apply)**

- Street friends
- Housed friends
- Family
- Church or spiritual/faith group
- Other **__________________**

**86. How do they help you?**

- Provide housing
- Provide food
- Provide transportation
- Access detox
- Access health care (addiction/mental health/physical health)
- Access counseling
- Access housing
- Access legal
- Access employment
- Access Transportation
- Talking through problems/emotional support
- Don’t know
- Refused to answer

**87. How often do you access your social supports?**

□ Yearly or less

□ Monthly or less

□ Weekly

□ Daily or almost daily

□ Don’t know

□ Refused

**88. If no, do wish you did?**

- Yes
- No
- Don’t know
- Refused to answer

***That concludes our survey. Thank you for coming in today! Do you have any question***
